# The Role of Tissue-resident γδ T Cells in Stress Surveillance and Tissue Maintenance

**DOI:** 10.3390/cells9030686

**Published:** 2020-03-11

**Authors:** Margarete D. Johnson, Deborah A. Witherden, Wendy L. Havran

**Affiliations:** Department of Immunology and Microbiology, The Scripps Research Institute, 10550 N. Torrey Pines Rd., La Jolla, CA 92037, USA; mdjohnso@scripps.edu (M.D.J.); hibarra@scripps.edu (W.L.H.)

**Keywords:** γδ T cell, tissue-resident, wound healing, damage repair, epithelial, adipose

## Abstract

While forming a minor population in the blood and lymphoid compartments, γδ T cells are significantly enriched within barrier tissues. In addition to providing protection against infection, these tissue-resident γδ T cells play critical roles in tissue homeostasis and repair. γδ T cells in the epidermis and intestinal epithelium produce growth factors and cytokines that are important for the normal turnover and maintenance of surrounding epithelial cells and are additionally required for the efficient recognition of, and response to, tissue damage. A role for tissue-resident γδ T cells is emerging outside of the traditional barrier tissues as well, with recent research indicating that adipose tissue-resident γδ T cells are required for the normal maintenance and function of the adipose tissue compartment. Here we review the functions of tissue-resident γδ T cells in the epidermis, intestinal epithelium, and adipose tissue, and compare the mechanisms of their activation between these sites.

## 1. Introduction

Very shortly after their initial identification, γδ T cells were described to have a unique distribution compared to αβ T cells. Early studies in mice, humans, and chickens demonstrated that γδ T cells preferentially home to tissues over lymphoid organs [1,2,3,4,5,6,7], and that this tissue localization of γδ T cells is dependent on Vγ chain expression. It should be noted that two different nomenclatures are in use for the murine Vγ chains, the Heilig and Tonegawa system [8] and the Garman system [9], which we use here. γδ T cells are found across a wide range of tissues including the skin, lungs, gut, genital tract, and adipose tissue [10,11]. Parabiosis studies have demonstrated a non-migratory phenotype for these tissue-resident γδ T cells in multiple tissues including the skin, gut, and adipose tissue [12,13,14]. In the mouse, dendritic epidermal T cells (DETC) express an invariant Vγ3Vδ1 receptor [15,16], γδ T cells in the epithelia of the tongue, uterus, and vagina express a Vγ4Vδ1 T cell receptor (TCR) with limited diversity [17], and γδ T cells of the intestinal epithelium (IELs) express receptors in which the Vγ5 chain pairs with a number of different Vδ chains [15,18]. These different γδ T cell compartments are not just divided in their tissue localizations, but also in the timing of their development in the thymus with T cells expressing different Vγ chains developing at different times [10,19,20] based on their proximity to the J segment [21]. Together, these features suggested a particularly important role for γδ T cells in the barrier tissues, as well as a potentially more limited diversity in antigen recognition than is seen for αβ T cells. This led to the idea that γδ T cells fill a functional niche between the innate and adaptive immune systems and form the “first line of defense” at barrier surfaces where they can rapidly respond to infection or insult [10,22,23]. While γδ T cells have the ability to recognize and respond to a variety of stimuli, they are particularly well situated for monitoring self-tissue for signs of stress. The majority of research on tissue-resident γδ T cells has focused on those residing in barrier tissues, but more recent work has indicated a role for γδ T cells in the maintenance of tissue outside of these sites as well. In this review we focus on the functions of three spatially distinct compartments of γδ T cells in maintaining the integrity and function of surrounding tissue: the clonal Vγ3Vδ1 DETC population in the epidermis, the primarily Vγ5+ population in the intestinal epithelium, and the Vγ4+ population in the adipose tissue. 

## 2. Epidermal γδ T Cells in Maintenance and Repair

### 2.1. Epidermal Structure and Function

The epidermis forms the barrier between the body and the outside world, playing a critical role in preventing water loss and providing protection from injury [24,25]. The epidermis is a stratified structure in which keratinocytes of the basal, proliferative layer are continually differentiating and moving outwards toward the stratum corneum, the topmost layer composed of dead corneocytes, in order to maintain homeostasis [24,26]. Keratinocytes account for over 90% of all epidermal cells [26], forming the major structural component of this organ and also playing a crucial role in initiating an inflammatory response to infection. Keratinocytes have the ability to sense pathogen-associated molecular patterns (PAMPs) through a number of Toll-like receptors (TLRs) and can respond with the production of a variety of inflammatory cytokines, anti-microbial peptides, and chemokines important for the recruitment of neutrophils and T cells [27,28].

The epidermis is also home to several types of immune cells including Langerhans cells and T cells. Langerhans cells are dendritic antigen presenting cells residing in the suprabasal epidermal layers that are involved in the initiation of both pro- and anti- inflammatory responses [27]. While the T cell population in the mouse epidermis is entirely comprised of DETC [15,16], both αβ and γδ T cells are present in the human epidermis, though at lower numbers than is observed in mice [29,30,31]. Less is known about the skin-resident γδ T cell population in humans, but evidence suggests that these cells primarily express receptors with Vδ1 chain usage [29,32,33,34] rather than the Vδ2 chain usage most common in peripheral blood. The population of epidermal-resident γδ T cells in both mice and humans is particularly well positioned to react rapidly to epidermal insult and injury, especially given that murine DETC are known to respond to stressed keratinocytes [35,36].

### 2.2. DETC Development and Trafficking to the Epidermis

DETC precursors are the first T cells to appear in the mouse thymus, developing in a defined window between embryonic day 13 and 18 [19,20]. At this time in development terminal deoxynucleotidyl transferase is not expressed in the thymus and rearrangement occurs with preference for the Vγ3 chain directly proximal to the J segment [21,37,38], resulting in a canonical invariant Vγ3Vδ1 TCR. Upon egress from the thymus, these cells home to the epidermis where they undergo homeostatic proliferation to maintain the epidermal compartment throughout the lifetime of the mouse [15,16]. The specific expression of the Vγ3Vδ1 TCR is not necessary for the development of an epidermal T cell population as γδ T cells expressing alternate TCRs are present in the epidermis of mice with mutations in the Vγ3 and Vδ1 genes, and αβ T cells populate the epidermis of TCRδ^-/-^ mice [39,40,41]. However, TCR signaling does appear to be important for the formation of the epidermal DETC compartment by driving the upregulation of receptors important for thymic egress and epidermal homing. During normal development, DETC precursors upregulate the skin homing receptor CCR10 and the sphingosine-1-phosphate receptor 1 (S1PR1) which is important for thymic egress [37,42]. Mice deficient for IL-2 inducible T cell kinase (ITK), a kinase downstream of TCR signaling, do not show CCR10 and S1PR1 upregulation during maturation [43]. These mice have delayed accumulation of DETC in the epidermis but do eventually develop a normal DETC compartment. This observation indicates that TCR signaling is important for the development of DETC precursors in the thymus, but dispensable for survival and proliferation in the epidermis. In addition to CCR10, the expression of CCR4 and ligands for both E-selectin and P-selectin are important for DETC homing, though they do not appear to play a role in normal thymic development [44]. 

Cytokine signaling has also been shown to play a role in development as DETC are absent in mice lacking either IL-15 [45] or IL-7 [46]. IL-7 plays a critical role in the induction of V-J recombination and IL-15 supports DETC proliferation and survival during development [45,47]. Another important factor in DETC development is the expression of *skint1* on thymic epithelial cells. Although the mechanism by which γδ T cells recognize *skint1* is unclear, Vγ3+ DETC are absent in the FVB-Taconic mouse strain in which a natural mutation in the *skint1* gene has arisen [48,49]. The expression of *skint1* during thymic development plays an important role in shaping DETC function by programming DETC precursors towards IFNγ production and away from IL-17 production [50]. While the precise mechanisms governing DETC development have not yet been fully elucidated, it is clear that a highly regulated array of overlapping and distinct signals are required for DETC maturation and epidermal homing. 

### 2.3. DETC Functions in Homeostasis and Damage Repair

Once in the epidermis, DETC take on their characteristic morphology in which long dendrites are spread out between neighboring keratinocytes where they are poised to recognize damage or infection [51,52]. Chodaczek et al. demonstrated that under steady-state these dendrites are primarily oriented towards the apical epidermis, where clusters of TCR appear to be engaged with keratinocyte-expressed ligand in long-lived immunological synapses [53]. The authors show that DETC exist in a state of preactivation under homeostatic conditions and are ready to rapidly respond to stress signals in neighboring cells. 

DETC are important for homeostatic maintenance of the epidermis, and in their absence the epithelial barrier is compromised. DETC-deficient mice exhibit increased transepidermal water loss when placed under dry housing conditions, demonstrating the importance of these cells in responding to environmental fluctuations [54]. Following TCR stimulation DETC have been shown to secrete insulin-like growth factor 1 (IGF-1), and in the absence of DETC mice show increased epidermal apoptosis that is reversed by IGF-1 administration [55]. In addition to their role in epithelial cell survival, DETC have the ability to secrete a number of chemokines including lymphotactin (LTN, XCL1), macrophage inflammatory protein (MIP-1α, CCL3), and CCL5, indicating a role in shaping the lymphocyte compartment of the murine epidermis [56]. Further evidence for DETC regulation of the lymphocyte compartment comes from work showing that the loss of Vγ3+ DETC results in αβ T cell-mediated cutaneous inflammation [57]. Together these studies demonstrate that a functional DETC population is critical for epidermal maintenance under homeostasis.

Beyond their role in maintaining the epithelial barrier at homeostasis, γδ T cells are key players in the wound healing response, and wound healing is delayed in both TCRδ^-/-^ and FVB-Taconic mice [58,59,60]. Following wounding, DETC retract their dendrites and begin secreting growth factors such as KGF-1, KGF-2 [29] and IGF-1 [55] that promote proliferation in neighboring keratinocytes. Additionally, DETC-produced KGF-1 and KGF-2 has been demonstrated to induce keratinocyte production of hyaluronan, a glycosaminoglycan that is an important component of the extracellular matrix and is involved in lymphocyte migration to sites of damage [61]. By this mechanism, DETC are ultimately able to mediate the recruitment of macrophages to the wound site, again exhibiting a role in shaping the lymphocyte compartment of the epidermis. A small subset of DETC are also able to produce IL-17A in response to wounding, which has been shown to contribute to barrier restoration by inducing proliferation and the production of anti-microbial peptides in surrounding keratinocytes [62]. From these experiments it is clear that DETC respond to epithelial damage on a number of fronts, orchestrating the proliferation of keratinocytes, infiltration of other leukocyte subsets, and clearance of pathogens to restore homeostasis. While less is known about the epidermal-resident T cell population in humans, both αβ T cells and γδ T cells in the human epidermis produce IGF-1 in response to wounding and activation of these cells results in more rapid wound closure in a skin-organ culture model of healing [29]. Interestingly, in contrast to what is seen in acute wounds, αβ T cells and γδ T cells isolated from human chronic wounds do not produce IGF-1 and are refractory to further stimulation [29] suggesting these cell populations as potential clinical targets in the treatment of non-healing wounds. 

γδ T cell activation in response to wounding relies on interactions with a number of different stress-induced signals expressed on keratinocytes. Although the ligand for the murine Vγ3Vδ1 TCR is unknown, it has been shown to be upregulated on keratinocytes following wounding by staining with a fluorescently labeled Vγ3Vδ1 TCR tetramer [35,63]. Furthermore, blocking TCR-ligand interactions with this soluble Vγ3Vδ1 TCR tetramer results in delayed healing [63] and while the αβ+ DETC that populate the epidermis of TCRδ^-/-^ mice are responsive to direct stimulation with ConA or anti-CD3, they are unresponsive to damaged keratinocytes [39]. Taken together, these studies show that the recognition of keratinocyte-expressed antigen by the Vγ3Vδ1 TCR is essential for the DETC-mediated wounding response.

**Figure 1:**
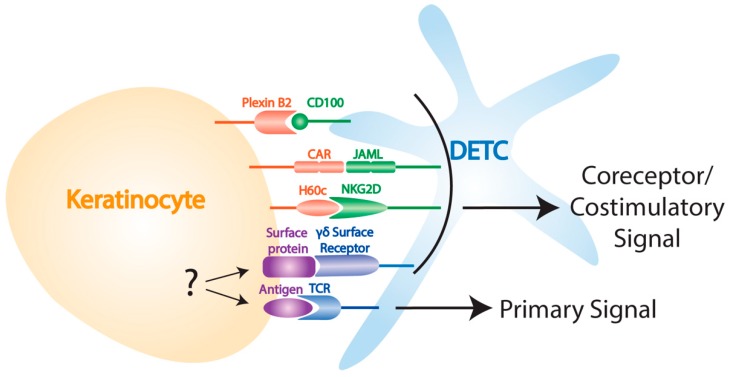
Ligands involved in DETC activation. Keratinocyte-DETC interactions are important for DETC activation post-wounding. Known interactions include CD100-plexin B2, JAML-CAR, NKG2D-H60c, and primary signaling through TCR following interactions with an unknown antigen.

In addition to signals through the TCR, mouse studies have demonstrated that DETC also rely on interactions with a number of other stress signals to recognize and respond to damaged self (Figure 1). Following wounding, keratinocytes upregulate their expression of the coxsackie and adenovirus receptor (CAR) which provides a costimulatory signal through the DETC-expressed junctional adhesion molecule-like protein (JAML) that is functionally similar to CD28/B7 costimulation in αβ T cells [64,65]. A costimulatory signal through NKG2D is also provided by H60c, an MHC class I-like molecule that is increased on keratinocytes following wounding [66,67]. While the DETC-expressed receptors recognizing skint proteins have not been defined, defects in the upregulation of these molecules in aged mice results in impaired DETC activation and wound healing [60]. Additionally, keratinocyte-expressed plexin B2 is important for initiating the DETC rounding response through ERK kinase and cofilin signaling following ligation with DETC-expressed CD100 [68]. In the absence of any of these interactions wound healing is delayed [64,66,68], and it thus appears that a number of overlapping signals are required for efficient DETC activation in the wound environment. While the reasons for this redundancy are not fully clear, it may be that a requirement for multiple danger signals helps to prevent DETC from becoming activated in the absence of damage. It is also possible that different ligands may interact with DETC at different times during the activation phase, helping to coordinate distinct aspects of the wound response. 

The DETC-mediated damage response is not limited to the wounding context, and these cells are known to respond to a variety of epidermal insults. TCRδ^-/-^ mice have increased susceptibility to models of cutaneous carcinoma, and DETC are able to kill skin carcinoma cells *in vitro* through an NKG2D sensitive mechanism [69]. Additionally, epidermal γδ T cells are activated in response to UVR in both mice and humans and TCRδ^-/-^ mice exhibit reduced levels of DNA repair following UVR exposure [70]. It should be noted that DETC can play a pathogenic role in epidermal homeostasis as well, and their presence is required for a contact hypersensitivity reaction in the DNFB mouse model [71]. DETC-mediated contact hypersensitivity is partially reversed through the blocking of NKG2D [72], indicating overlap between DETC activation in this pathogenic pathway and activation in DETC-mediated tissue repair. Overall, it is clear that DETC are essential mediators in the epidermis, maintaining tissue homeostasis at steady state and orchestrating the damage response to a range of insults.

## 3. Intestinal Epithelial γδ T Cells in Maintenance and Repair

### 3.1. Structure and Function of the Intestinal Epithelium

The intestinal epithelium is comprised of a single layer of cells which serve to provide a barrier between the luminal contents and the lamina propria. This structure coordinates the uptake of water and nutrients and enables crosstalk between the immune system and luminal microbes. In the small intestine the epithelium is organized into protruding villi and invaginating crypts, while in the colon villi are not present and the epithelium forms a flatter surface. The intestinal epithelium is characterized by constant turnover and is the most rapidly renewing tissue in the body [73,74]. Stem cells in the bottom of the crypts continuously produce progenitor cells which differentiate and travel outwards until reaching the top of the apical surface where they undergo apoptosis and slough off into the lumen [73,74,75]. Effective barrier function requires constant and coordinated proliferation and migration of epithelial cells in a process that is critical to maintaining gut homeostasis. Dysfunction in the epithelium can result in inflammation driven by bacterial invasion of the mucosal surface, and has been linked to the development of inflammatory bowel disease (IBD) [76]. 

The intestinal epithelium is primarily composed of enterocytes, which are responsible for the absorption of water and nutrients, but also includes mucus secreting goblet cells and hormone-secreting enteroendocrine cells [73,74,75]. Also residing in the epithelium are specialized cell types that coordinate the host interactions with gut microbiota and pathogens. These include chemosensory tuft cells, which provide protection against helminths, Paneth cells (in the small intestine), which secrete anti-microbial peptides, and M cells, which are involved in the uptake of luminal antigen for presentation to immune cells [75,77]. 

In addition to these epithelial cell components, a significant number of intraepithelial lymphocytes (IELs) reside in the intestinal epithelium, comprising around 10% of the cells in this tissue in mice [78], and around 15-20% in humans [79]. The IEL compartment is often subdivided into two categories: the type a or “induced” IELs expressing αβ TCR with CD8αβ or CD4 and the type b or “natural” IELs expressing either γδ TCR or αβ TCR without CD8αβ (often expressing CD8αα) [80,81]. Type a IELs are MHC-restricted, functionally very similar to αβ T cells in the lymphoid organs, and become activated after encountering antigen in the periphery. Conversely, type b IELs reside almost exclusively in the epithelium, acquire an activated phenotype during development, and appear to be biased towards self-antigen recognition [80,82]. The proportion of γδ IELs varies over development, comprising around 80% of the IEL compartment in suckling mice and dropping to around 20% in older animals [83]. γδ IELs are present in both the small intestine and colon where they have been found to form approximately 30% and 10% of the murine IEL compartment, respectively [84]. Despite this lower prevalence in the colon, γδ IELs are widely distributed throughout the intestinal epithelium where they play a key role in maintaining its barrier function. 

### 3.2. γδ IEL Development and Trafficking to the Intestinal Epithelium

Murine γδ IELs primarily express Vγ5+ TCRs, but unlike in the case of the invariant Vγ3Vδ1 TCRs expressed by DETC, Vγ5 can pair with multiple δ chains [18,85]. These cells populate the intestinal epithelium around birth, and continue to be generated in the adult thymus [86]. It has long been suggested that γδ IELs may be able to develop extrathymically, but this remains a subject of controversy [80,87]. While IEL populations are present in athymic mice [83,88] it appears that thymic development is the main, and possibly exclusive, pathway of development in euthymic mice [89]. Like all γδ T cells, γδ IELs rely on IL-7 signaling for development as this cytokine is required for V-J recombination [47]. However, the role of IL-15 in the development of γδ IELs diverges from its role in DETC development. While in DETC development, IL-15 signaling appears to impact the proliferation and survival of precursors [45], in γδ IEL development IL-15 signaling controls the accessibility of the Vγ5 gene segment for rearrangement by inducing chromatin modifications [90]. Additionally, in the absence of IL-15 signaling the γδ IEL compartment is significantly reduced [91,92], and this cytokine has also been described to play a role in the differentiation [93], proliferation [94], survival [95,96], and motility [97] of γδ IELs.

Interactions between γδ IEL-expressed CCR9 and the chemokine CCL25 have been shown to be important for γδ IEL homing to the small intestine. CCL25 is highly expressed in both the thymus and intestinal epithelium [98], and mice deficient in either CCR9 or CD25 have reduced numbers of γδ IELs [99,100]. It is interesting to note that CCR9 expression appears to be negatively correlated with TCR-ligand interactions in the thymus, indicating a preference for the trafficking of antigen naïve γδ IELs to the intestine [100]. Additional evidence that positive selection through TCR signaling is not required for the development of a γδ IEL compartment comes from the observation that these cells are still present in G8 TCR transgenic mice on a β_2_ microglobulin-deficient background [101,102]. The G8 TCR recognizes the MHC class I antigens T22 and T10 which are absent in this MHC class I-deficient mouse strain, so any γδ IELs present in these mice must develop in the absence of TCR interaction with cognate ligand.

### 3.3. γδ IEL Functions in Homeostasis and Epithelial Damage Repair

While DETC are primarily sessile within the epidermis, γδ IELs actively migrate throughout the intestinal epithelium [103] by an occludin-dependent mechanism [104]. This strategy allows γδ IELs to engage in close contacts with a number of epithelial cells, which would otherwise be impossible given the monolayer structure of the intestinal epithelium. The migratory ability of γδ IELs has been shown to be important for γδ IEL-mediated defense against both *Salmonella typhimurium* and *Toxoplasma gondii* in mice [105]. DETC and γδ IELs reside in close proximity to microorganisms in the skin and intestine, respectively, and function in the control of both host microbiota and invading bacteria [106]. While the overall impact on γδ T cell-mediated barrier maintenance is unknown, studies in germ-free and antibiotic-treated mice have suggested that the microbiota can influence the functions of γδ T cells in these tissues, and the presence of commensals is required for the normal distribution and migration of γδ IELs [106,107]. γδ IELs have been described as having an “activated but resting” phenotype in which cytotoxic genes are expressed at steady state alongside the expression of inhibitory receptors [108,109], a combination that may allow a rapid response rate while maintaining control over effector function at homeostasis.

Although γδ IELs have been shown to play a role in host defense against pathogens, it has been suggested that their main function is in maintaining the homeostasis and barrier integrity of the intestinal epithelium [80,110]. One mechanism of protection appears to be through limiting the response of other IELs to various insults [80,111]. γδ IELs have been shown to dampen the inflammatory response in mouse models of IBD with αβ T cell-mediated pathology [112,113], and to prevent damage due to an overactive pathogen response by murine type a αβ IELs [114]. Interestingly, it was demonstrated that γδ IELs expressing the inhibitory receptor NKG2A isolated from patients with celiac disease suppressed the cytotoxic phenotype of αβ IELs in vitro through the secretion of TGF-β [115]. Although the mechanisms are not yet well understood *in vivo*, these studies suggest that γδ IELs play an important role in regulating the immune responses of type a IELs. γδ IELs also play a more direct role in maintaining the integrity of the intestinal epithelium by promoting epithelial cell proliferation. Mice that are deficient in γδ T cells show reduced intestinal epithelial cell turnover at steady state, though no such defect is observed in mice deficient in αβ T cells [116]. It has also been shown that γδ IELs, but not αβ IELs, are able to produce KGF upon stimulation [117] indicating a likely mechanism for this γδ IEL-mediated epithelial cell proliferation. Furthermore, altered epithelial cell proliferation in mouse models of villus atrophy and hypertrophy were later demonstrated to depend on γδ IEL production of KGF [118], providing a direct link between γδ IEL production of KGF and epithelial proliferation. 

The ability of γδ IELs to mediate epithelial cell function is particularly important when the barrier is disrupted due to damage or infection. γδ IELs have been demonstrated to provide protection in a DSS model of intestinal injury and repair, localizing at sites of damage and inducing epithelial cell proliferation through the production of KGF [119]. In addition to exerting control over proliferation, γδ IELs also play a role in regulating epithelial barrier permeability during bacterial infection by controlling the organization of tight junction proteins [120]. Together, these studies highlight the ability of γδ IELs to maintain the intestinal epithelial barrier through direct regulation of neighboring epithelial cells. The role of γδ IELs in gut inflammation and repair in humans is not well understood, although it has been suggested that decreased numbers of γδ T cells in the ilium of preterm infants with necrotizing enterocolitis may contribute to the pathogenesis of this disease [121]. While the proportion of γδ IELs have been shown to increase in patients with celiac disease [122,123,124], there is contradictory evidence of both increased and decreased numbers of γδ IELs in the inflamed mucosa of IBD patients [125,126]. The variable results of these human studies highlight the need for additional research on the γδ IEL response over the course of inflammatory diseases in the gut in order to resolve their potential causal and protective roles.

**Figure 2:**
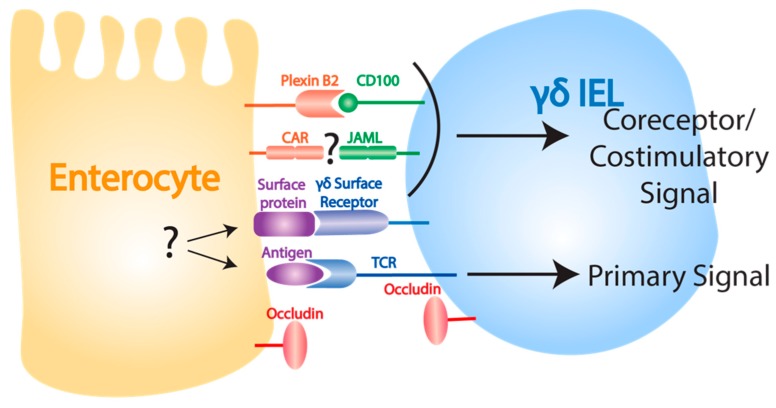
Ligands involved in γδ IEL function. The mechanisms behind γδ IEL activation following intestinal epithelial damage are less well understood than the mechanisms underlying DETC activation in the skin, but plexin B2-CD100 interactions are known to play a role. Intestinal epithelial cells and γδ IELs express CAR and JAML, respectively, but whether these interactions provide a costimulatory signal following epithelial damage is unknown. The role of antigen recognition by TCR and the presence of other costimulatory signals also remain unknown. Occludin expressed by both γδ IELs and intestinal epithelial cells is important for γδ IEL migration.

Despite a clear role for γδ IELs in epithelial barrier maintenance and repair, less is understood about how resident γδ T cells are activated in response to epithelial damage in the gut than in the skin. Interestingly, while signaling through the TCR is important for DETC activation in response to epithelial insult in the skin [63], γδ IELs in some instances have been shown to have the ability to recognize pathogens independently of their TCR [127]. However, recent studies suggest that similar coreceptors may be required for γδ T cell activation the skin and gut (Figure 2). One such study demonstrated that γδ IEL-mediated protection from dextran sulfate sodium (DSS)-induced epithelial damage was dependent on CD100-plexin B2 interactions [128]. Additionally, while the effect of this receptor-ligand pair is unknown in the gut, JAML and CAR are expressed on murine γδ IELs [64] and intestinal epithelial cells [129], respectively. Given the expression of these molecules in the gut, and the observation that costimulation through JAML *in vitro* induces proliferation in γδ IELs [64], it is possible that JAML-CAR costimulatory interactions may be important in the γδ T cell-mediated damage response in the gut as they are in the skin. γδ IELs have also been shown to respond to infection through the recognition of epithelial-cell expressed MyD88, providing further evidence for the importance of epithelial cell crosstalk in γδ IEL activation [127,130]. While much remains to be discovered about how γδ IELs recognize and respond to epithelial damage, it appears possible that these cells may share activation features with their DETC counterparts.

## 4. Adipose Tissue-resident γδ T Cells in Inflammation and Obesity

### 4.1. Functions and Organization of Adipose Tissue

Adipose tissue forms the largest endocrine organ in humans, making up approximately 20-50% of an individual’s total body weight [131]. While once considered primarily to be an inert energy storage tissue, it is now clear that adipose tissue performs a wide array of functions ranging from shock absorption and thermal insulation to tissue repair, thermogenesis, and the secretion of anti-microbial peptides and cytokines [132,133]. Adipose tissue can be subdivided into two main categories: brown adipose tissue (BAT) which is primarily involved in thermogenesis and most prevalent at the fetal and infant stages, and white adipose tissue (WAT) which comprises the vast majority of adipose tissue in adults. WAT can be further divided into the visceral WAT (vWAT) including mesenteric, gonadal, and omental WAT, and subcutaneous WAT (sWAT) located under the skin [133]. While there is evidence that the sWAT fraction plays a metabolically protective role, the vWAT fraction has been linked to obesity and metabolic disorders [134,135].

Adipocytes are the major cell type in adipose tissue, comprising up to 90% of tissue volume but only approximately 50% of the cellular content due to their large size [131,136]. The considerable stromal-vascular fraction of adipose tissue contains a wide range of immune cells including endothelial cells, fibroblasts, macrophages, eosinophils, mast cells, innate lymphoid cells (ILCs), and lymphocytes [137]. Lymphocytes account for 10-15% of the stromal-vascular fraction and include B cells, αβ T cells, invariant natural killer (iNKT) cells, T regulatory (Treg) cells, and γδ T cells [137,138]. Under normal conditions adipose tissue immune cells perform both defensive and homeostatic functions, but shifts in this compartment towards an inflammatory phenotype during obesity have been well documented [136,137,138]. The mechanisms behind this pro-inflammatory shift are still being elucidated, but an increased number of macrophages and a bias towards M1 polarization seems to play a role [137,139,140,141]. The T cell compartment also appears to be involved in obesity-related inflammation although the various influences of different subsets remains unclear. While CD8+ T cells are known to increase during obesity [142,143] the timing of their increase is not well understood. Evidence has been presented supporting a model in which CD8+ T cell accumulation precedes and drives macrophage accumulation [144,145] as well as a model in which CD8+ T cell accumulation follows and is driven by macrophage accumulation [146]. In contrast to the pro-inflammatory CD8+ T cell population, Tregs and iNKT cells are both enriched in lean adipose tissue and decreased during obesity [147,148,149]. While it is clear that obesity-related inflammation in the adipose tissue involves the complex interplay of a number of cell types, the contributions of different T cell subsets are still being resolved.

### 4.2. The Role of γδ T Cells in Obesity and Adipose Tissue Inflammation

γδ T cells, mostly expressing Vγ4+ TCRs, form a tissue-resident population in the murine adipose tissue [12], but an understanding of their functional significance is only just beginning to emerge. These cells are enriched in adipose tissue, forming between 5-15% of the total T cell compartment [11,12] and are increased during high fat diet (HFD)-induced obesity [11,150]. This increase in γδ T cell numbers on a HFD was initially shown to contribute to macrophage accumulation, inflammation, and insulin resistance [150] indicating a pathogenic role for γδ T cells in obesity-related inflammation. However, a later study identified a crucial role for murine adipose tissue-resident γδ T cells in maintaining the adipose Treg population [12]. The authors found that IL-17A-producing γδ T cells were the driving factor in promoting stromal-cell production of IL-33 which in turn promotes the maintenance of the adipose Treg population. Additionally, peroxisome proliferator-activated receptor β (PPAR-β) overexpressing mice which exhibit lower numbers of αβ T cells and higher numbers of γδ T cells are protected from weight gain, inflammation, and insulin resistance on a high fat diet [151]. Together these studies demonstrate that γδ T cells play an important role in shaping both pro- and anti- inflammatory T cell populations in adipose tissue and highlight the need for additional research in this area. While little is known about the impact of obesity on γδ T cell function in the adipose tissue, obesity has been shown to impact γδ T cells in other compartments. In murine models of obesity, DETC have an impaired ability to regulate keratinocyte homeostasis and wound healing [152,153]. Additionally, both αβ and γδ IELs exhibit lower numbers and reduced expression of CD103 and CCR9, proteins that are important for IEL retention in the intestinal epithelia [154]. These studies suggest that an obese state may have a detrimental effect on the functions of tissue-resident γδ T cells and highlight the need for additional research on the adipose tissue-resident population. Intriguingly, a recent study by Goldberg *et. al.* has demonstrated that γδ T cells are initially increased in the adipose tissue of mice on a ketogenic diet but decreased in the long-term following ketogenic diet-induced obesity [155]. Further research is needed to understand the circumstances and functional differences that drive adipose γδ T cells to accumulate or decrease in number during systemic metabolic shifts.

**Figure 3:**
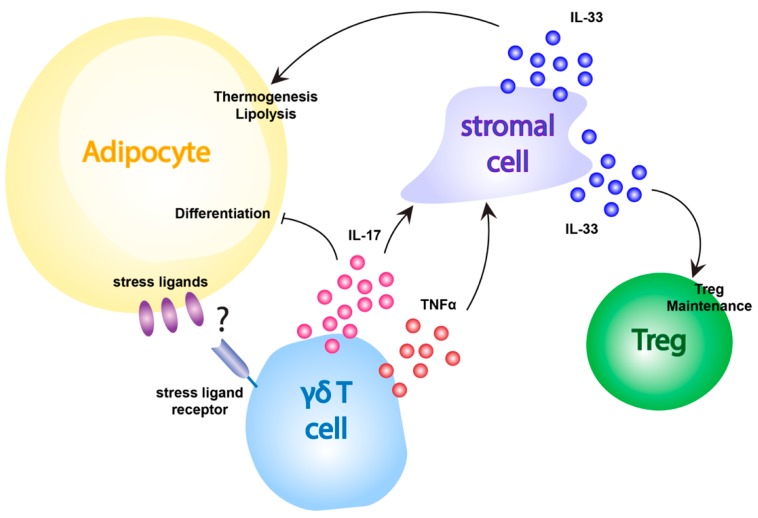
Adipose γδ T cell activation and function. Adipose tissue-resident γδ T cells affect adipocyte metabolism and Treg maintenance through the release of IL-17 and TNFα which induce IL-33 production in stromal cells. This IL-33 acts to promote thermogenesis and lipolysis pathways in adipocytes of the BAT and inguinal WAT, and to maintain the Treg compartment in the adipose tissue. Additionally, γδ T cell-produced IL-17 appears to act directly on adipocytes to inhibit differentiation. How γδ T cells become activated to perform these functions in unclear, but could rely on the recognition of stress ligands expressed by adipocytes.

γδ T cells also appear to have more direct effects on adipocyte function, in addition to shaping other T cell populations in the adipose tissue (Figure 3). For example, adipose tissue-resident γδ T cells produce IL-17A, which in turn drives IL-33 production by adipose tissue stromal cells that promotes lipolysis and thermogenesis in both BAT and inguinal WAT [12]. Additionally, γδ T cell-produced IL-17 has been shown to negatively regulate adipogenesis and protect against obesity in mice [156]. Recent work from Hu *et. al.* [157] demonstrates that γδ T cell-produced IL-17F plays a key role in the innervation of adipose tissue, and provides an additional link between adipose tissue-resident γδ T cells and thermogenesis. It is possible that this IL-17 pathway may serve as a negative feedback mechanism to control the production of excess adipose tissue, but at this point virtually nothing is known about how γδ T cells in adipose tissue are activated. Interestingly, the production of IL-17 has been shown to increase following HFD-induced obesity, leading the authors to propose that this may be the result of γδ T cell recognition of obesity-associated stress ligands expressed on adipocytes [156]. This would suggest a similar mechanism of γδ T cell activation may be in play in adipose tissue as is present in the skin, gut, and other barrier sites. However, the ligands involved in γδ T cell surveillance of adipose tissue are currently unknown and additional work is needed to understand how γδ T cells monitor and respond to stress in this unique environment.

## 5. Conclusions

γδ T cells form a highly conserved branch of the adaptive immune system, having existed alongside αβ T cells since the two cell populations first emerged [158,159]. While the functional delineations between these groups continue to be clarified, a primary role for γδ T cells as surveyors of self-tissue was suggested almost concurrently with their discovery. Data collected over the ensuing three decades has largely supported this model, and it is clear that γδ T cells are well adapted to sensing a variety of stresses in a range of tissues. γδ T cells are particularly important in the continuously renewing barrier tissues of the skin and gut where they play a critical role in recognizing damage and promoting proliferation in the surrounding epithelial tissue. More recently, a role for γδ T cells has begun to emerge in the adipose tissue [12,156], a compartment structurally and immunologically distinct from the barrier tissues in which γδ T cells have long been recognized to be important. However, given the developing view that cellular stress and inflammation are increased during obesity, it makes sense that γδ T cells could play a role in surveilling and shaping this tissue compartment as well. 

Critical to understanding how γδ T cells function in maintaining tissue homeostasis is clarifying the mechanisms by which they are activated in response to damage and stress. Studies on the skin and gut resident γδ T cell populations have demonstrated a key role for the engagement of a number of different costimulatory molecules for this activation. Interestingly, while there is more known about γδ T cell activation in response to damage in the skin than in the gut, there appears to be some overlap in these signals with both populations responding to epithelial-expressed plexin B2 through the semaphorin CD100 [68,128]. In addition, the costimulatory protein CAR is expressed by keratinocytes [64], intestinal epithelial cells [129], and adipose tissue [160], raising the possibility that JAML-CAR interactions may be a shared mechanism for activating tissue-resident γδ T cells across multiple compartments. Given the clinical importance of epithelial damage in the skin and gut, as well as the emerging threat of obesity-related disorders, a better understanding of how γδ T cells are activated to perform their homeostatic functions across these tissues is greatly needed.
